# Venus reawakening

**DOI:** 10.1038/s41467-026-72086-4

**Published:** 2026-05-11

**Authors:** Julia Maia, Maxence Lefèvre

**Affiliations:** 1 Institute of Space Research, German Aerospace Center (DLR), Berlin, Germany; 2 LATMOS/IPSL, Sorbonne Université, UVSQ Université Paris-Saclay, Centre National de la Recherche Scientifique, Paris, France

**Keywords:** Planetary science, Planetary science

## Abstract

Despite similarities in size and density to Earth, Venus followed a radically different evolution, becoming a hot, dry world. Here we review key properties of the planet and discuss how forthcoming missions may explain its puzzling evolutionary history.

## The divergent twin

At the dawn of modern astronomy, observations of Venus’ phases provided key evidence for the heliocentric theory^1^. By the mid-20th century, Venus was recognized as Earth’s slightly smaller twin, comparable in size and mass but hidden beneath a dense, opaque atmosphere. The lack of surface information stimulated speculation that Venus hosted oceans and life^2^.

This view changed dramatically through two major advances: radio astronomy and planetary exploration. Ground-based radar observations revealed that Venus radiates at ~600 K^3^, interpreted as an extremely hot surface caused by a runaway greenhouse effect [e.g., ref. ^4^]. NASA’s Mariner 2 mission^5^, humanity’s first successful interplanetary spacecraft, confirmed Venus’ high temperatures^6^. In 1967, the Soviet Venera 4 probe performed in situ atmospheric measurements, revealing a dense atmosphere composed of 95% CO_2_^7^.

**Fig. 1:**
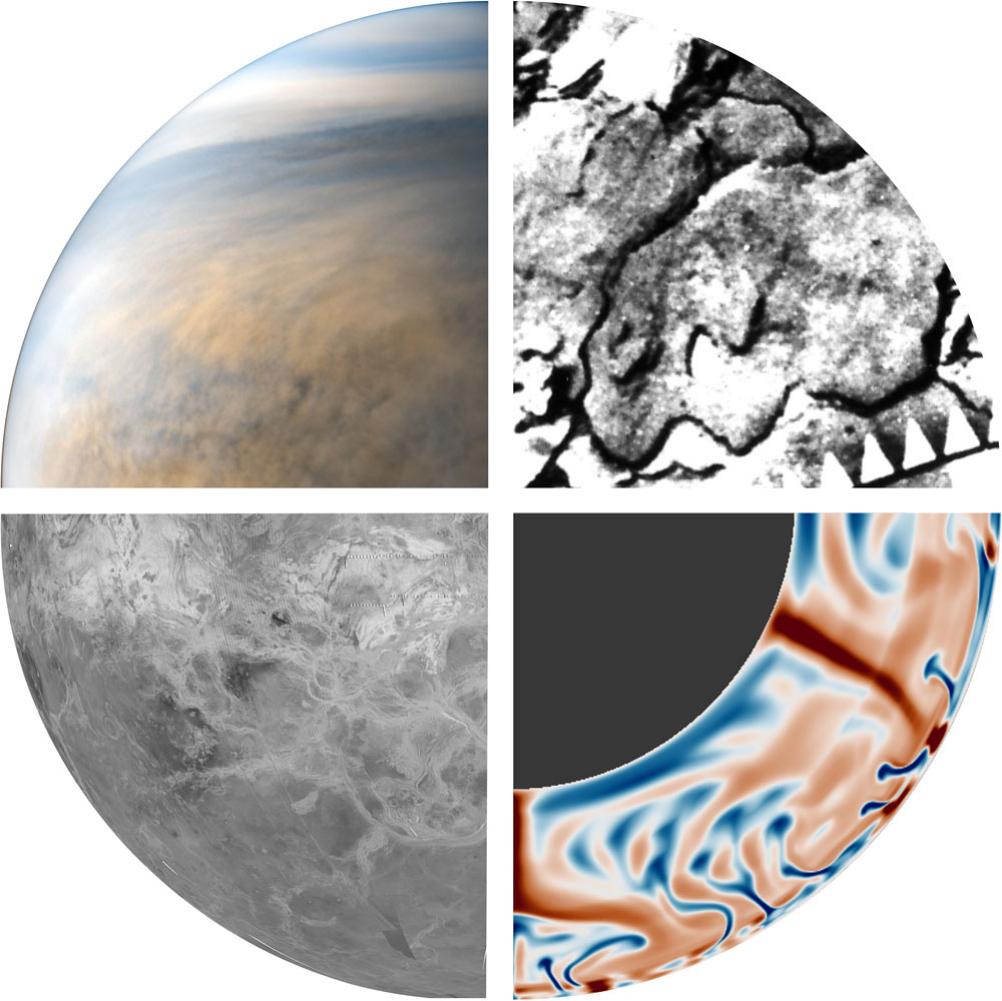
Atmosphere, surface, and interior of Venus. Top-left: Venus' cloud tops observed in the ultraviolet by the Akatsuki cameras (credit: JAXA/ISAS/DARTS/Kevin M. Gill). Top-right: Portion of a process panorama image acquired by the Venera 14 lander, showing Venus' surface rocks (prepared by Stephen Paul Meszaros). Bottom-left: Magellan SAR images mosaic of parts of Ovda Regio, Thetis Regio, and Artemis Corona, shown in orthographic projection. Bottom-right: Geodynamic simulation of Venus' mantle, with red indicating high-temperature anomalies (upwellings) and blue indicating low-temperature regions (downwellings), courtesy of Carianna Herrera.

Subsequent Venera missions achieved numerous milestones. Venera 7 performed the first soft landing on another planet and measured surface temperatures near 740 K^8^. Later landers transmitted the first surface images (Fig. 1) and analyzed local compositions, revealing basaltic materials similar to terrestrial basalts^9,10^. In parallel, NASA’s Pioneer Venus Orbiter (PVO) provided a global perspective of the planet. PVO produced the first global topographic map and found no evidence for plate tectonics^11^, while gravity data suggested active mantle convection^12,13^. Together, these observations demonstrated that Venus operates under a fundamentally different geodynamic regime [e.g., ref. ^14^], a conclusion reinforced by the absence of an intrinsic magnetic field^15,16^.

These discoveries underscored the need for global, high-resolution radar observations, achieved in the 1990s by the Magellan mission (Fig. 1). Using synthetic aperture radar (SAR) and altimetry, Magellan mapped 98% of Venus’ surface^17^. The mission revealed a surface dominated by volcanism, with over 80,000 volcanic edifices^18^. The largest edifices cluster at broad topographic rises, interpreted as a mantle hotspot. Venus also hosts highly tectonized highlands that may be continental analogues with felsic compositions, potentially signalling a water-rich past^19^. Moreover, hundreds of circular tectono-volcanic structures known as coronae were identified [e.g., ref. ^20^], some exhibiting characteristics consistent with localized subduction^21,22^.

Magellan further revealed an unusual crater population: only ~900 impact craters, minimally modified and almost randomly distributed, implying a uniformly young surface aged 250–750 Myr^23^. Two competing resurfacing hypotheses emerged. One invoked continuous, stochastic resurfacing^24–26^. The other proposed a catastrophic global resurfacing event ~500 Myr ago, followed by a sharp decline in geological activity^27,28^. The catastrophic model became predominant, reinforcing the long-standing assumption that Venus is presently geologically inactive [e.g., refs. ^29,30^]. This perception, combined with growing emphasis on Mars exploration, contributed to reduced investment in Venus missions.

The atmosphere of Venus is also fundamental to understanding the planet’s evolution. In the 21st century, Venus exploration has focused on the atmosphere through Venus Express by ESA (2006–2014) and Akatsuki by JAXA (2015–2025), which made observations across a wide range of wavelengths.

A defining atmospheric feature is Venus’ ~20 km-thick global cloud layer^31^. While appearing bright and uniform in visible light, ultraviolet observations (Fig. 1) revealed strong contrasts and evolving large-scale patterns^32,33^. Spectroscopy and polarimetry indicated a composition of sulfuric acid mixed with water^34,35^, later confirmed by in situ measurements from the Pioneer Venus Large Probe, which also identified three distinct particle size distribution^36^, from submicron to few microns, one order of magnitude smaller than typical Earth cloud particles^37,38^. The dark UV markings are caused by strong absorption from SO_2_, SO, and at least one still unidentified species, which controls the upper-cloud deposition of incoming solar flux. While sulfur compounds were long favored candidates^39^, recent studies suggest iron chlorides as a plausible absorber^40,41^, though their abundance remains poorly constrained.

Ultraviolet cloud tracking also revealed wind speeds exceeding 100 m s^−1^ at 70 km altitude, circling the planet in 4–5 days^42^, far faster than Venus’ 243-day rotation period^43^. This atmospheric super-rotation is characterized by angular momentum transport balance between the mean meridional circulation and transient waves^44^. Akatsuki further demonstrated the dominant role of thermal tides in low-latitude super-rotation^45^.

These missions also provided key constraints on atmospheric chemistry and Venus’ potential habitability. Measurements of the deuterium-to-hydrogen (D/H) ratio below the clouds revealed values over 100 times Earth’s^46,47^. Venus Express detected a tenfold increase in D/H above the clouds^48^, possibly linked to differential photodissociation of H_2_O and HDO. Such enrichment is often interpreted as evidence for a lost primordial ocean^49^, though volcanic outgassing and cometary delivery may also contribute^50^. In addition, decadal variations in SO_2_ at cloud-top altitudes measured by Venus Express and Pioneer Venus have been proposed as indicators of ongoing volcanic outgassing^51^.

Venus Express also detected surface emissivity anomalies associated with volcanic features^52,53^, interpreted as recent, unweathered lava flows^54,55^. Together with SO_2_ variability in the clouds^51^ and surface changes observed in Magellan radar data^56,57^, the observations seem to indicate a geologically active Venus^58^ and potentially challenge the catastrophic resurfacing hypothesis. Understanding Venus’ resurfacing history is therefore essential for reconstructing its geological evolution, internal heat loss, and interior-atmosphere volatile exchanges, processes that ultimately shape its climate and potential past habitability.

## Understanding Venus’ evolutionary pathways

Venus is increasingly understood as a dynamic planet in which interior, surface, and atmospheric processes are intimately linked. Today, A central question guides Venus research: why did Venus follow a radically different evolution from Earth? Addressing this requires reconstructing Venus’ history from accretion^59,60^ and magma-ocean solidification^61,62^ through its geodynamic^63^ and climate evolution^64^. Advances in numerical modeling have enabled exploration of plausible evolutionary pathways despite limited observational constraints.

Solid-state mantle convection is the primary mechanism by which Venus cools its interior (Fig. 1). Mantle dynamics govern magma generation and tectonic deformation, and the interplay between these processes defines the planet’s geodynamic regime and heat-loss efficiency. After Magellan, Venus was widely interpreted to operate in an episodic-lid regime, in which a largely stagnant lithosphere undergoes periodic large-scale mobilization driven by internal stress accumulation^29,65–67^. This scenario aligns with the catastrophic resurfacing interpretation. More recent geodynamic models, particularly those incorporating melting processes, have identified an alternative regime: the plutonic-squishy lid^68^. In this case, extensive intrusive magmatism weakens the lithosphere, enabling surface mobilization and localized recycling. This regime is consistent with Venus’ inferred lithospheric thermal structure^69^ and supports the continuous resurfacing scenario. Determining Venus’ geodynamic regime and heat-loss mechanisms remains a primary objective of Venus research.

A key discriminator between these regimes is the current level of geological activity. While there is growing evidence for ongoing volcanism^56,58^, the global volcanic flux remains poorly constrained, and the extent of active tectonic deformation is still uncertain. Quantifying volcanic activity is also essential to constrain the volatile exchange between the interior and the atmosphere. Volatiles stored in the interior and released through volcanism have supplied Venus’ atmosphere throughout its history, requiring coupled models of interior and atmospheric evolution^70–72^.

State-of-the-art climate models provide two opposing scenarios for Venus’ early evolution. Some propose that early liquid water stabilized a temperate climate, later terminated by catastrophic resurfacing and runaway greenhouse warming^73^. Others argue that nightside cloud formation produces a net warming that inhibits surface condensation, yielding a persistently hot and dry planet^74,75^. Resolving this debate ultimately requires observational constraints. Improved measurements of the D/H ratio and noble gas isotopes are essential for reconstructing Venus’ atmospheric evolution^76^. Monitoring atmospheric minor species is also critical for constraining present-day volcanic degassing and major sinks^64^, as well as identifying the nature of the enigmatic UV absorber^31^, and therefore differentiating climate evolution from climate variabilities. Evidence of past liquid water may also be preserved in Venus’ crust, as large volumes of silica-rich rocks indicate hydrous melting on Earth^77^; however, similar compositions may form under anhydrous conditions on Venus^78^.

## A pivotal decade for Venus exploration

For many years, the Venus community’s motto has been “we need better data”. Indeed, resolving the planet’s fundamental mysteries requires improved observations across all frontiers, i.e. new missions. A turning point came in 2021 with the selection of three Venus missions. NASA approved VERITAS^79^, an orbiter focused on geology and geophysics, and DAVINCI^80^, an atmospheric descent probe. ESA subsequently selected EnVision, which will investigate the coupled evolution of the atmosphere, surface, and interior^81^. Together, these missions will transform our understanding of Venus.

High-resolution radar imaging and topography will enable reconstruction of Venus’ geological history. Gravity measurements will constrain lithospheric structure and heat flow, as well as the size, density, and physical state of the core, providing insights into accretion and long-term thermal evolution^82,83^. Global surface emissivity measurements will map the presence of felsic material^84^ and identify thermal anomalies associated with active volcanism. Combined with atmospheric monitoring of minor species, especially below the clouds for the first time, these datasets can constrain Venus’ volcanic flux^85,86^. In parallel, in situ measurements of isotopic ratios and noble gases will be essential for reconstructing degassing history, atmosphere-surface interactions, and long-term water loss^80^.

The scientific importance of these missions is clear, and their selection revitalized a community that had been shrinking for years. However, at the time of writing, budgetary pressures at NASA has led to uncertainties for the schedule and/or continuation of the three Venus missions. Prolonged uncertainty comes with a risk of loss of expertise, notably from the Magellan generation, and affects scientists and engineers whose work depends on the schedule and continuity of these missions.

While the community works to ensure these missions move forward, other agencies are also turning their attention to Venus. India has announced plans for its first Venus mission, China and Russia have presented ambitious exploration concepts, and a privately funded atmospheric mission is expected to launch this year. Simultaneously, the community is actively defining the next generation of Venus exploration, including long-duration landers^87^, aerial platforms^88–91^, novel approaches to seismology^92^, and atmospheric sample return concepts^93^.

The importance of this exploration extends beyond Venus itself. As discoveries of Earth-sized worlds continue to grow, understanding why planets that begin with broadly similar properties can follow radically different evolutionary paths becomes essential. Determining the conditions that drive this divergence is fundamental to assessing planetary habitability. Venus represents a critical benchmark: without understanding its evolution, our ability to interpret Earth-like exoplanets and to define the limits of habitability remains incomplete.
